# Awake intubation with videolaryngoscopy and fiberoptic bronchoscope

**DOI:** 10.1002/ccr3.5274

**Published:** 2022-01-11

**Authors:** Shusuke Utada, Hiromu Okano, Hiroshi Miyazaki, Shoko Niida, Hiroshi Horiuchi, Naoya Suzuki, Tsuyoshi Otsuka, Ryosuke Furuya

**Affiliations:** ^1^ Department of Critical Care and Emergency Medicine National Hospital Organization Yokohama Medical Center Yokohama Japan

**Keywords:** awake intubation, difficult airway, fiberoptic bronchoscope, videolaryngoscopy

## Abstract

By combining video laryngoscopy and fiberoptic bronchoscopy, awake intubation can be performed more safely.

## Graphical abstract

The simultaneous display of bronchoscope and laryngoscope images on a single screen allows for safer conscious tracheal intubations.

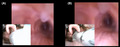

## 1 CASE PRESENTATION

Intubation with a fiberoptic bronchoscope is an important technique.^1^ Although the safety of video laryngoscopy versus bronchoscopy for tracheal intubation in patients with difficult airways has been investigated, sufficient evidence is lacking.^2^

**FIGURE 1:**
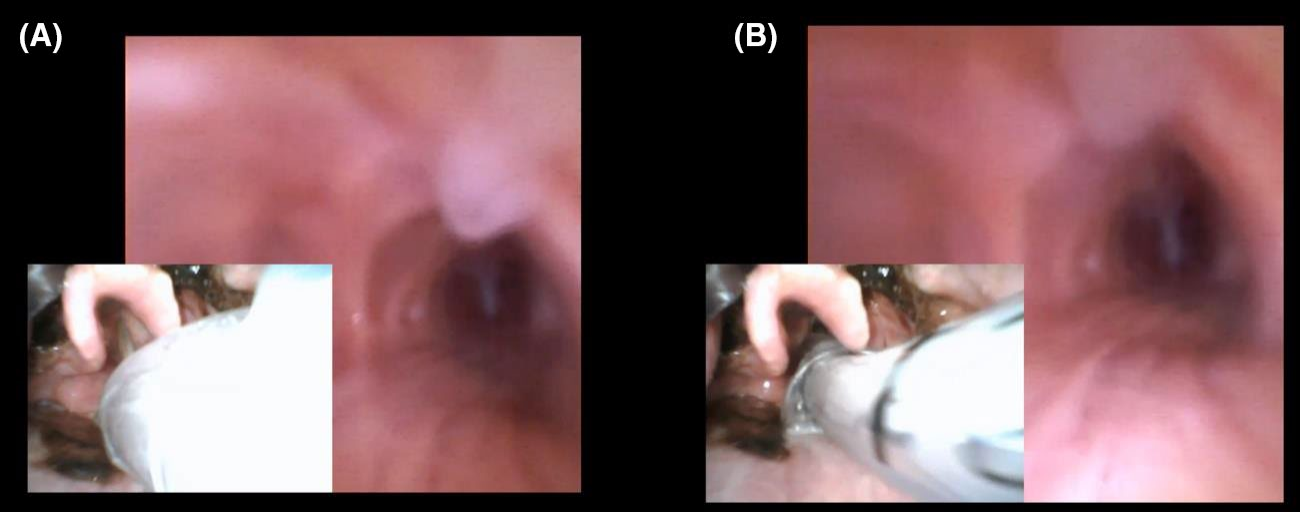
Bronchoscope and video laryngoscope images during intubation. The bronchoscope and video laryngoscope images are displayed on the same screen. In both images, the bronchoscope had already penetrated into the trachea. (A) The glottis was closed. (B) The intubation tube was inserted through the bronchoscope while the glottis was open

Fiberoptic intubation is disadvantageous because it does not allow sufficient observation when passing through the glottis. A multifaceted view of video laryngoscopy is necessary to ensure safety during awake intubation using a bronchoscope. We used the Glidescope 1 Core™ 10 VL/LoPro S1 (Verathon Medical 50 Canada ULC, Burnaby, BC, Canada), which allows for the insertion of a tracheal tube while the glottis is open (Video S1, Figure 1).

## CONFLICT OF INTEREST

Nothing to declare.

## AUTHOR CONTRIBUTIONS

SU wrote the first draft. HO, HM, SN, HH, NS, TO, and RF critically revised it, and all authors read and approved the final version of the manuscript.

## ETHICAL APPROVAL

The article does not contain any studies with human participants or animals.

## CONSENT

Written consent was obtained from the patient.

## Supporting information

Video S1
